# Influence of Vegetation Structure on Lidar-derived Canopy Height and Fractional Cover in Forested Riparian Buffers During Leaf-Off and Leaf-On Conditions

**DOI:** 10.1371/journal.pone.0054776

**Published:** 2013-01-31

**Authors:** Leah Wasser, Rick Day, Laura Chasmer, Alan Taylor

**Affiliations:** 1 Pennsylvania State University, University Park, Pennsylvania, United States of America; 2 National Ecological Observatory Network, Boulder, Colorado, United States of America; 3 Department of Ecosystem Science and Management, Pennsylvania State University, University Park, Pennsylvania, United States of America; 4 Cold Regions Research Centre, Wilfrid Laurier University, Waterloo, Ontario, Canada; 5 Department of Geography, Earth and Environmental Systems Institute, Pennsylvania State University, University Park, Pennsylvania, United States of America; NASA Jet Propulsion Laboratory, United States of America

## Abstract

Estimates of canopy height (H) and fractional canopy cover (FC) derived from lidar data collected during leaf-on and leaf-off conditions are compared with field measurements from 80 forested riparian buffer plots. The purpose is to determine if existing lidar data flown in leaf-off conditions for applications such as terrain mapping can effectively estimate forested riparian buffer H and FC within a range of riparian vegetation types. Results illustrate that: 1) leaf-off and leaf-on lidar percentile estimates are similar to measured heights in all plots except those dominated by deciduous compound-leaved trees where lidar underestimates H during leaf off periods; 2) canopy height models (CHMs) underestimate H by a larger margin compared to percentile methods and are influenced by vegetation type (conifer needle, deciduous simple leaf or deciduous compound leaf) and canopy height variability, 3) lidar estimates of FC are within 10% of plot measurements during leaf-on periods, but are underestimated during leaf-off periods except in mixed and conifer plots; and 4) depth of laser pulse penetration lower in the canopy is more variable compared to top of the canopy penetration which may influence within canopy vegetation structure estimates. This study demonstrates that leaf-off lidar data can be used to estimate forested riparian buffer canopy height within diverse vegetation conditions and fractional canopy cover within mixed and conifer forests when leaf-on lidar data are not available.

## Introduction

Forested riparian buffers provide numerous ecosystem services including pollutant filtration, wildlife habitat, stream flow mitigation and temperature mediation and thus maintain the ecological integrity of aquatic and terrestrial ecosystems [1]–[4]. However, their often narrow and fragmented spatial configuration make them vulnerable to disturbance which can impede buffer ecological function and yield broadscale ecological degradation [5]–[8]. Riparian vegetation canopy height and fractional canopy cover are two frequently measured indicators of buffer and associated stream integrity that are incorporated into monitoring initiatives (e.g. EMAP) around the world [9]–[11]. These metrics can be used to predict key biophysical stream and terrestrial attributes including stream temperature, detritus availability, available wildlife habitat and biomass [12]–[18].

Identifying and monitoring change in riparian buffer structure over broad geographic extents is important for understanding disturbance impacts on riparian forest dynamics, associated ecological consequences and the efficacy of restoration efforts. Yet detection and quantification of riparian forest change over time is difficult [19]. Detection methods, including *in situ* plot sampling [17], [20] and classification of multi-spectral satellite imagery or aerial photography [4], [21]–[23], introduce a compromise between spatial resolution (grain) and area extent [19], [24], [25]. Frequent plot sampling, while necessary, is time and cost intensive limiting inventory geographic extent and riparian corridor widths are often more narrow than the grain of a multi-spectral, multi-temporal remote sensing pixel (e.g. Landsat) limiting detection [26], [27]. While some spectral remote sensing methods can be used to effectively assess vegetation condition over broad geographic areas for relatively low costs [23], these techniques are unable to accurately characterize important within canopy structural attributes.

Discrete return airborne Light Detection and Ranging (lidar) provides a link between *in situ* field and spectral remote sensing techniques used to detect and quantify riparian buffer characteristics, especially in areas where buffers are narrow and fragmented. Lidar systems measure the three-dimensional characteristics of vegetation structure by sampling the canopy, understory and ground surface using reflected light from a rapidly emitted laser pulse. Within canopy reflections or ‘returns’ allow for estimation of forest structure and composition including canopy and understory height, stand basal area and fractional cover [28]–[36]. Interpolation of discrete return lidar can be used to create seamless datasets that can in turn, be used to continuously map three-dimensional forested riparian vegetation conditions at high spatial resolutions. Lidar thus offers the unique opportunity to bridge the gap between horizontal resolution, sampling geographic extent and three-dimensional vertical structure that exists between spectral RS detection methods and plot data [9], [14], [37], [38]. Despite these benefits, lidar surveys flown during maximum leaf-flush conditions, commissioned for vegetation detection are not always available for large geographic regions, whereas leaf-off lidar surveys, often commissioned for terrain generation, have expanded significantly (e.g. PAMAP, USGS National Map) with many parts of North America, Australia, and Europe being recently surveyed.

Increased lidar survey coverage provides an opportunity for land managers, ecologists and others to use lidar data that were previously collected for other purposes to monitor vegetation structure [39]. Yet, the accuracy of canopy height and fractional cover estimates using data flown during periods when vegetation is not in maximum leaf-flush conditions may be compromised. Further, the degree to which estimates accurately represent canopy height and fractional cover will vary with species type and lidar survey timing within the phenologic cycle. However, the utility of lidar flown during leaf-off conditions has been demonstrated in some forest types. For example, Naesset [40] and Orka et al. [41] demonstrated that leaf-off lidar data can estimate some biophysical properties within Scandinavian forests (stem density, basal area and average canopy height), while Brandtberg et al. [42]; Brandtberg [43]; Kim et al. [44] and others have found that leaf-off lidar data can be used to classify vegetation in North American forests. There is a need to assess the accuracy of existing lidar data in estimating vegetation structure within a variety of ecosystems if researchers wish to utilize it in support of broad-scale vegetation inventory and assessment.

Thus, the objectives of this study are to: 1) compare leaf-on and leaf-off lidar estimates of canopy height and fractional canopy cover with plot measurements within riparian buffers of varying vegetation types to assess whether leaf-off lidar data are adequate for characterizing forested riparian buffer structure and 2) determine if the vegetation types (and associated leaf and branch structure) typical of northeastern (USA) riparian forests influence leaf-off and leaf-on lidar estimate accuracy.

## Materials and Methods

### Study Area

**Figure 1:**
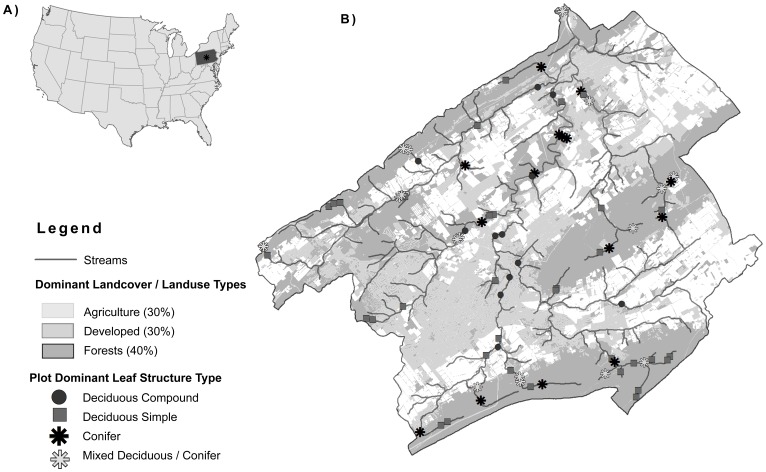
Spring Creek Watershed: A) Context map of the United States identifying the location of the study area, Spring Creek Watershed, located in the center of Pennsylvania; B) Landuse/landcover map of Spring Creek illustrating streams, landuse and mensuration plot locations examined in the study (Landuse Data Source: Centre County Planning Commission, 2006).

The study area, Spring Creek Watershed, is located within the Ridge and Valley province of Central Pennsylvania, and is part of the Chesapeake Bay watershed (Lat. 40.818°N, Long. 77.841°W). Landcover in Spring Creek is dominated by forest (40%), agriculture (30%) and urban/suburban development (30%) within an elevation range of 200 m–730 m (Fig. 1). Riparian buffers within lowland watershed valleys are narrow, surrounded by agricultural or developed landuse types. Vegetation within these lowland riparian forests is dominated by a host of species including black walnut (*Juglans nigra*) and maple (*Acer spp.*) with multi-flora rose (*Rosa multiflora*) common in the understory. Riparian buffers within upland ridges are largely undisturbed, surrounded by large tracts of open forest. Ridge top riparian buffers are ephemerally wet and are dominated by upland vegetation including Oaks (*Quercus spp.*), Maples (*Acer spp.*) and Mountain Laurel (*Kalmia latifolia*) understory. Forested buffers within lower ridge elevations have regularly saturated floodplains dominated by Eastern Hemlock (*Tsuga canadensis*), Black Birch (*Betula nigra*) and Maple (*Acer spp.*) with Rhododendron (*Rhododendron spp.*) understory.

### Lidar Data Collection and Processing

Leaf-off lidar data, part of a larger operational acquisition for the state of Pennsylvania, known as Pennsylvania Map (PAMAP), were acquired for the study area between April 26–29^th^, 2006. Although leaf-off lidar data were collected at the end of April, leaf flush had not yet occurred as determined by coincident photos taken at varying elevations throughout the watershed. Leaf-on lidar data, were collected between June 15–18^th^, 2007 (Table 1). Lidar data were acquired using different flight parameters. These differences in flight parameterization were accounted for by comparing leaf-on and leaf-off lidar vegetation structure estimates within conifer plots where needles are retained year round.

**Table 1:** Leaf-off and leaf-on lidar survey specifications.

	Leaf-off Lidar	Leaf-on Lidar
Flight Dates	April 26–29, 2006	June 15–18, 2007
Sensor	Optech ALTM 3100	Leica ALS50
Scan Angle	+/−21.5 degrees	+/−20 degrees
Average Flying Height	1,900 m	900 m
Pulse Repetition Frequency	40.6 kHz	100 kHz
Returns Collected	First, only, last	First, intermediate, last, only
Average Footprint	0.57 m	0.18 m
Point Spacing	1.4 m	0.8 m

Lidar data were classified into ground and non-ground returns using TerraScan (TerraSolid, Helsinki, Finland). Leaf-on lidar data were thinned to be comparable to the lower resolution, leaf-off lidar dataset (1.4 m point spacing, 2 returns). Data thinning utilized a gridded approach with random removal of laser pulse returns. For both lidar datasets, ground only, vegetation (>1.3 m, to coincide with digital hemispherical photography, DHP, height) and all (ground+vegetation) point clouds were extracted for the entire watershed and individual plots. A 1.8 m resolution digital surface model (DSM) of the maximum (canopy plus ground) elevation and a leaf-off lidar derived digital elevation model (vertical accuracy = 0.38 cm, DEM) were used to generate a canopy height model (CHM) representing the difference between the DSM and the DEM. To compare gridding methods, the CHM was created using both interpolation and non interpolation methods. Inverse distance weighted interpolation was used for DSM generation as a commonly used interpolation procedure for lidar data [45], [46]. Lidar data were also gridded without interpolation using the max point cloud height value for each 1.8 m cell. Lidar CHMs were used to derive the mean height value within each 11.3 m radius plot for comparison with measured plot average canopy height (*H*). Lidar decile and quartile percentile height values were extracted from the frequency distributions of laser pulse returns greater than 2 m above ground level [40], [47]. Lidar percentile height values were compared with *H* to identify lidar best estimates. Finally, fractional canopy cover was estimated by the ratio of the total number of canopy returns (>1.3 m) to all returns [31].

### Mensuration Plots

Permission was obtained from the appropriate landowners, for access to all forest plots that occurred on private lands. Vegetation canopy and understory structure including canopy height, canopy base height, diameter at breast height (DBH), fractional canopy cover and species type were measured and recorded during leaf-on conditions in eighty 400 m^2^ (r = 11.3 m) circular plots during July–August, 2010 (Table 2). A total of 80 plots were selected according to vegetation type (deciduous, conifer and mixed) and height (8–30 m) within a 100 m buffer of either side of watershed streams using a random stratified sampling design (Fig. 1). Plot centers were geographically located using a Trimble GeoXH differential GPS unit (Trimble Inc, GeoExplorer, California, USA) with horizontal accuracy better than 0.3 m. GPS points were collected for 30 minutes or more for each plot using a one second logging rate. The average horizontal accuracy of final plot location points was 0.2 m with a range of less than 0.1 m to 0.3 m. Diameter at breast height (DBH) was recorded for all stems with a height greater than 2 m and DBH greater than 0.07 m. Stem apex height and canopy base height were measured using a LaserAce Hypsometer (MDL, Aberdeen, Scotland) with a vertical accuracy of 0.05 cm or better [48]. All saplings (stems greater than 2 m in height and less than 0.07 m DBH) were recorded by species for each 1 m height class. For each plot, mean canopy height and canopy base height were calculated using dominant and intermediate stems (stems which contribute to the top of the canopy). To account for vegetation growth that occurred between the 2006/2007 lidar surveys and 2010 field mensuration, measured canopy stem heights were reduced in Forest Vegetation Simulator (FVS, US Forest Service, Fort Collins, CO, USA). FVS uses plot specific data including stem density, species, height, and DBH to predict annual growth; data were calibrated for vegetation located within the North East United States. The mean height adjustment per plot was 0.24 m per year (σ = 0.05 m, Table 3). Growth adjustments did not significantly influence regression R^2^, RMSE values or between group differences detected in canopy height estimates.

**Table 2:** Summary of average plot characteristics within deciduous, conifer and mixed vegetation types.

Plot Vegetation Type	N	Mean Canopy Height (m)		Basal Area (m^2^)		Mean # Stems	Conifer (%)	Canopy FC	Elevation Range (m)	Dominant species
		Range	Mean	Range	Mean					
All Plots	80	9.3–30.3	21.2	0.1–2.6	1.4	8–38	0–96%	0.60–0.92	*220–545*	*59 species recorded.*
**Dominant Leaf Structure Type**										
Deciduous Simple	35	9.3–27.4	20.5	0.1–1.9	1.0	9–30	<15%	0.60–0.92	*280–545*	*A. rubrum, P. serotina, Q. rubra*
Deciduous Compound	12	12.9–21.4	17.5	0.3–1.4	0.9	11–23	<10%	0.69–0.92	*230–340*	*J. nigra, F. pennsylvanica, G. tricanthos*
Conifer	9	16.5–29.9	24.8	1.7–2.6	2.1	10–38	>90%	0.75–0.89	*254–507*	*T. canadensis, Pinus spp*
Mixed Conifer/Deciduous	24	13.7–30.3	22.8	0.8–2.6	1.8	8–34	30–90%	0.80–0.91	*223–532*	*T. canadensis, A. rubrum, A. saccarum*

N = number of plot samples.

**Table 3:** Adjusted height values per year in meters for all plots and by plot vegetation leaf structure type.

Adjusted Height Values	Leaf-off vs. Measured	
Plot	Mean	σ Mean
All Plots	0.25	0.05
Decid. Simple	0.24	0.06
Decid. Compound	0.24	0.03
Mixed Decid/Conifer	0.22	0.06
Conifer Needle	0.26	0.06

Riparian vegetation within the watershed is diverse with 59 species representing three dominant leaf-structure types (deciduous simple, deciduous compound broadleaf and conifer needle) recorded during field mensuration. Conifer species, (e.g. *T. canadensis*), have dense needles that are retained year round compared to deciduous vegetation which has a less dense leaf structure. Conversely, deciduous compound species (e.g. *J. nigra*) tradeoff woody lateral branches for leafy biomass yielding a more open leafless branch structure compared to deciduous simple leaved canopies with increased lateral branch structure [49]–[52]. It is known that species specific structural characteristics including leaf structure and orientation can influence laser reflections [53] however, dense vegetation did not allow for the isolation of species specific groups in plot classification. Therefore, final plot classification represented fundamental, dominant leaf-structure types (determined by basal area) for a total of four groups: 1) deciduous simple, 2) deciduous compound, 3) mixed deciduous/needle, and 4) conifer needle.

### Digital Hemispherical Photography

Fractional canopy cover (FC) per plot was estimated from gap fraction (1–gap fraction) using digital hemispherical photography (DHP) taken with a Canon EOS Rebel XS digital SLR (Canon, NY, USA) with a Sigma Circular Fisheye Lens (Sigma, NY, USA). Photographs were taken during diffuse sky conditions at a height of 1.3 m, 11.3 m from the plot centre, along four cardinal directions (N, S, E, and W) and at the plot center [38], [54]. Images were processed using DHP software (Version 4.6, S. Leblanc, Canada Centre for Remote Sensing) [54]. Plot FC was estimated using annulus rings 1–3, 1–4, 1–5 and 1–6 (mean zenith angles 13.5°, 18°, 22.5° and 27° respectively). DHP has an estimated average gap fraction accuracy of +/−10–20% when compared to other methods (e.g. TRAC, LAI-2000) [55].

### Statistical Analysis

Leaf-off and leaf-on lidar estimates of average canopy height (H) and fractional canopy cover (FC) were compared to plot measurements using linear regression. Best fit relationships were those where lidar estimates most closely represented plot measurements as determined by regression slope and intercept closest to a one to one relationship (near unity). To assess affects of vegetation type on lidar estimates, mean differences (*D*) were calculated between lidar estimates and *in situ* measurements for each plot and compared using analysis of variance (ANOVA). The influence of vegetation condition (leaf-off vs. leaf-on) on the distribution of laser returns throughout the canopy was measured in two ways. The coefficient of variation (*Cv*) was calculated for all non-ground leaf-off and leaf-on lidar points within each plot to estimate the vertical spread of within canopy laser response heights. Mean differences were also calculated between leaf-on and leaf-off lidar data for all decile and quartile percentile height values to estimate variation in penetration at the top, within and towards the bottom of the canopy. Leaf-on vs. leaf-off Cv and percentile mean difference values were compared both for all plots and between plots stratified by vegetation type using ANOVA. Histograms illustrating the frequency distribution of laser pulse returns throughout the canopy were also compared. To account for the influence of lidar flight parameterization, leaf-off and leaf-on lidar derived metrics were compared using ANOVA within conifer dominated plots (>90% conifer) where year-round needle retention removes the influence of vegetation condition. It was assumed that differences, attributed to lidar flight configuration, detected within these plots will propagate to all other forest plots [40].

## Results

### Differences in Leaf-on vs. Leaf-off Lidar Canopy Height Estimates Compared with Measured

**Figure 2:**
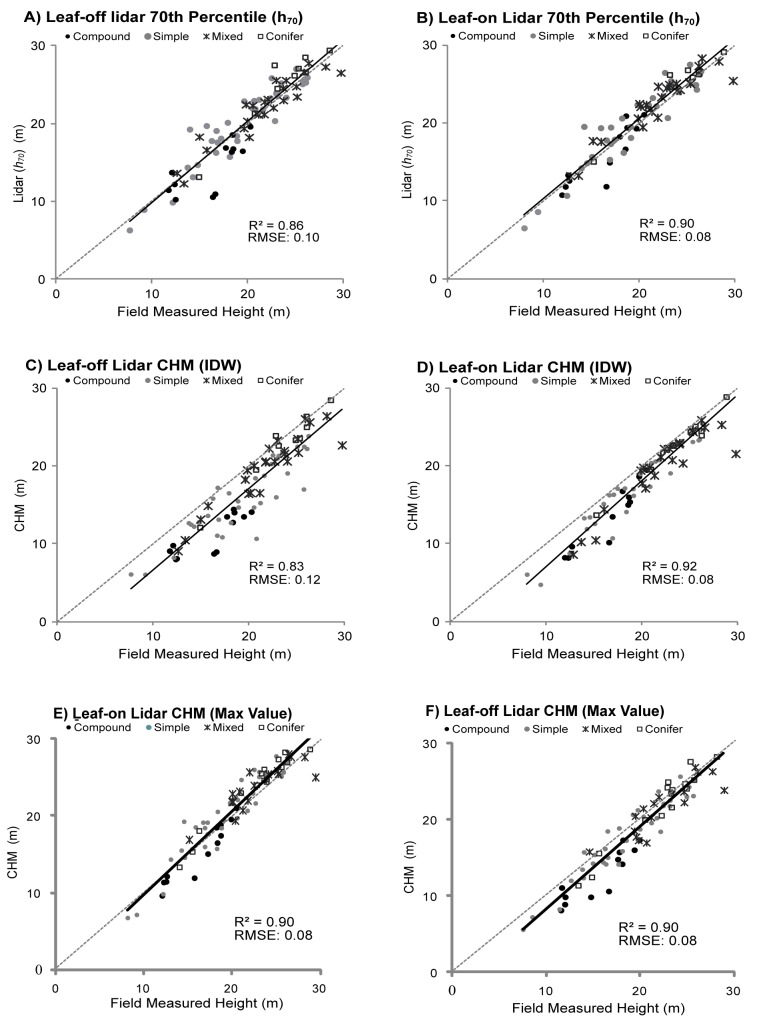
Regression relationships between measured average canopy height (H) and lidar values using: a) Leaf-off lidar 70^th^ percentile; b) Leaf-on lidar 70^th^ percentile; c) Leaf-off lidar IDW Interpolated CHM; d) Leaf-on lidar IDW Interpolated CHM; e) Leaf-off lidar Max Height CHM; f) Leaf-on lidar Max Height CHM.

Canopy heights estimated using: a) the percentile distribution of laser pulse returns within the canopy (Fig. 2A, 2B), b) IDW interpolation gridded canopy height model (CHM, Fig. 2C, 2D), and c) the max height gridded CHM (Fig. 2E, 2F) were compared to plot measurements. The 70^th^ percentile height value (*h*_70_) most closely related to measured canopy heights (*H*) with strong correspondence between *h*_70_ and *H* observed in leaf-on conditions (R^2^ = 0.90); leaf-off *h*_70_ relationships with *H* were similarly strong (R^2^ = 0.86). Mean differences between *h*_70_ and H were small on average in all plots in both leaf-on (D = 0.29 m) and leaf-off (0.48 m) conditions. CHM values also correlated well with *H. H* derived from the non-interpolated CHMs (using the max height) were most similar to field measured values (*D_Lon_* = −0.65 m, *D_Loff_* = −1.01 m). CHMs using interpolation methods underestimated measured canopy heights by a larger margin compared to *h*_70_ values in both leaf-off (*D* = −2.94 m) and leaf-on (*D* = −2.01 m) conditions. For all plots, statistically significant differences were not detected when comparing leaf-off with leaf-on lidar CHM (D = 1.16 m–2.19 m) or percentile height values (D = 0.4, Table 4).

**Table 4:** Mean differences in meters (Lidar - measured plot average height) for all plots and plots stratified by vegetation type using both percentile and CHM lidar height estimates.

CHM Values Interpolation	Leaf-off Lidar - Measured		Leaf-on Lidar - Measured		Leaf-on – Leaf-off	
Plot	*D*	σ (*D*)	D	σ (*D*)	D	σ (*D*)
All Plots	−2.9	2.3	−2.0	1.6	1.2	2.2
Decid. Simple	−3.4^A^	1.5	−1.8^B^	1.5	1.8^A^	2.3
Decid. Compound	−5.1^B^	1.7	−3.3^A^	1.5	2.1^A^	2.2
Mixed Decid/Conifer	−2.1^BC^	1.9	−2.1^AB^	1.9	0.2^B^	1.5
Conifer Needle	−0.9^C^	1.2	−1.1^B^	0.7	0.0^B^	1.2

CHM Values Max Height Grid	Leaf-off Lidar – Measured		Leaf-on Lidar - Measured		Leaf-on – Leaf-off	
Plot	*D*	σ (*D*)	*D*	σ (*D*)	*D*	σ (*D*)
All Plots	−1.0	1.8	0.7	1.7	2.2	1.0
Decid. Simple	−0.9^A^	1.5	0.8^A^	1.7	2.3^A^	1.0
Decid. Compound	−3.0^B^	1.5	−1.1^B^	1.3	2.4^A^	1.2
Mixed Decid./Conifer	−0.7^A^	1.9	1.0^A^	1.8	2.2^A^	1.0
Conifer Needle	−0.1^A^	1.4	1.3^A^	1.0	1.4^A^	1.0

70^th^ Percentile Values	Leaf-off Lidar – Measured		Leaf-on Lidar - Measured		Leaf-on – Leaf-off	
Plot	*D*	σ (*D*)	*D*	σ (*D*)	*D*	σ (*D*)
All Plots	0.3	2.0	0.5	1.7	0.4	1.1
Decid. Simple	0.7^A^	1.8	0.7^A^	1.8	0.2^B^	0.7
Decid. Compound	−1.8^B^	2.2	−0.7^A^	1.8	1.4^A^	1.6
Mixed Decid./Conifer	0.4^A^	1.6	0.6^A^	1.5	0.5^AB^	1.0
Conifer Needle	1.4^A^	1.7	0.9^A^	1.0	−0.3^B^	1.2

Superscripts (A,B,C) identify post-hoc Tukey test results. Means with the same superscripted letter are not statistically different (P = 0.05).

### Influence of Vegetation Type on Lidar Canopy Height Estimates

Riparian vegetation type influenced the accuracy of lidar percentile (*h*_70_) and CHM derived estimates of canopy height. Leaf-off and leaf-on lidar CHM and percentile estimates were most similar to H within conifer plots (D = −0.9–1.4 m, Table 4). The interpolated lidar CHM underestimated H by a larger margin (compared to the non interpolated CHM) within all plots. However, the non-interpolated leaf-on lidar CHM (CHM_max_) and lidar *h*_70_ both overestimated H by a small margin (0.9 m–1.3 m) in deciduous simple, conifer and mixed plots (excluding deciduous compound plots). In deciduous compound plots, lidar underestimated H by the largest margin using CHM (D = 3.3 m–5.1 m), CHM_max_ (D = 1.1 m–3.0 m) and percentile (*h*_70_, D = 0.7 m–1.8 m) methods. Regression relationships were also weaker within deciduous compound plots, particularly in leaf-off conditions using both CHM (R^2^ = 0.71–0.74, Table 5) and percentile methods (R^2^ = 0.59, Table 4). Interestingly, vegetation condition did not have a significant impact on top of the canopy height estimates within plots containing deciduous simple leaved and mixed (deciduous simple/conifer) vegetation. In these plots, close correspondence was found between leaf-off and leaf-on datasets although *h*_70_ overestimated (0.4–0.7 m) whereas CHM values underestimated *H* (1.8–3.4 m). CHM_max_ values were within ±0.9 m of field measurements. The influence of sensor and flight altitude on canopy height estimates as evidenced by differences between leaf-off vs. leaf-on CHM (D = 0.03 m) and *h_70_* (D = −0.25 m) values within conifer plots was small. Slightly larger differences were observed between leaf-off and leaf-on lidar *H* estimates when using CHM_max_ (D = 1.41).

**Table 5:** Regression relationships interpolated lidar CHM (CHM_interp_) vs. H, non-interpolated lidar CHM (CHMMax) vs. H and h70 vs. H for all plots and for plots stratified by leaf structure type.

CHM Interp Values	Leaf-off vs. Measured		Leaf-on vs. Measured	
Plot	R^2^	RMSE	R^2^	RMSE
All Plots	0.83	0.12	0.92	0.08
Decid. Simple	0.77	0.12	0.93	0.07
Decid. Compound	0.71	0.09	0.90	0.08
Mixed Decid./Conifer	0.86	0.08	0.84	0.09
Conifer Needle	0.96	0.04	0.97	0.03

CHM Max Values	Leaf-off vs. Measured		Leaf-on vs. Measured	
Plot	R^2^	RMSE	R^2^	RMSE
All Plots	0.90	0.08	0.90	0.08
Decid. Simple	0.91	0.11	0.90	0.07
Decid. Compound	0.80	0.09	0.91	0.08
Mixed Decid./Conifer	0.74	0.08	0.74	0.09
Conifer Needle	0.94	0.06	0.96	0.04

70^th^ Percentile Values	Leaf-off vs. Measured		Leaf-on vs. Measured	
Plot	R^2^	RMSE	R^2^	RMSE
All Plots	0.86	0.10	0.90	0.08
Decid. Simple	0.89	0.09	0.88	0.09
Decid. Compound	0.59	0.14	0.78	0.11
Mixed Decid/Conifer	0.87	0.07	0.87	0.07
Conifer Needle	0.91	0.07	0.94	0.04

RMSE values are standardized relative to the mean for each vegetation type.

### Differences in Leaf-on vs. Leaf-off Lidar Fractional Cover Estimates Compared with Measured

**Figure 3:**
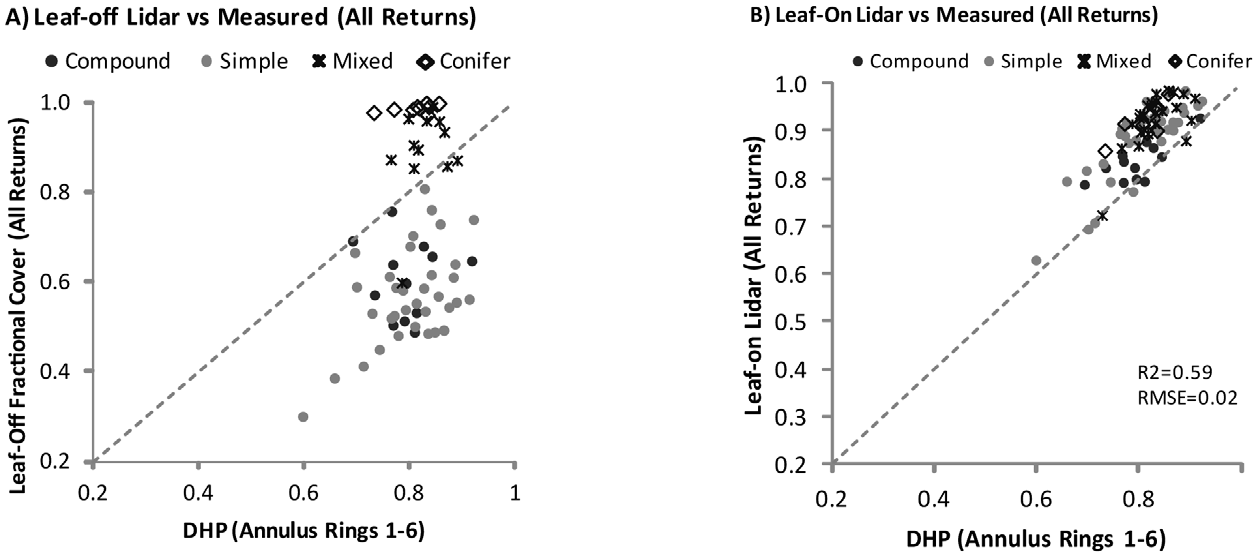
Relationships between leaf-off and leaf-on lidar FC estimates and measured FC using digital hemispherical photography. Axes are scaled differently to allow for best representation of the data. Regression relationships not shown for leaf-off lidar plot as data were not normally distributed precluding a statistically significant relationship.

Within all plots, fractional cover estimated using digital hemispherical photography (FC_DHP_) related well with leaf-on lidar estimates (FC_LOn_) using annulus rings 1–6 (R^2^ = 0.59, RMSE = 0.02) and was within 8% of measured values (Figure 3B). As expected, within all plots, leaf-off lidar FC values (FC_LOff_) underestimated measured fractional cover (FC_DHP_) and thus no relationship was found (Fig. 3A). In leaf-off conditions, it was evident that: 1) pulses most often yielded two returns given dense riparian vegetation, 2) first returns almost always represented canopy vegetation, and 3) first and only returns and last returns almost always represented the ground.

### Influence of Vegetation Type on Lidar Fractional Cover Estimates

Relationships between FC_LOn_ and FC_DHP_ were influenced by vegetation type, however, between plot differences were ranged from 4% to 11% (Range FC_diff_ = 0.04–0.11) of measured values (Table 6). FC_LOn_ estimates were most similar to FC_DHP_ within plots dominated by deciduous compound leaved vegetation whereby FC_LOn_ underestimated FC_DHP_ by 4% (σ = 4%) on average. In comparison, larger differences were observed between FC_LOn_ and FC_DHP_ in conifer, deciduous simple and mixed plots where differences ranged from 7 to 11%. While leaf-on lidar overestimated FC_DHP_ within plots of varying vegetation types, leaf-off lidar underestimated FC_DHP_. As expected, the largest differences between FC_LOff_ and FC_DHP_ were observed in plots dominated by deciduous simple (D = −19%) and compound (D = −24%) plots while smallest differences were observed within conifer (*D* = 6%, σ = 8%) and mixed plots (*D* = 8%, σ = 10%). The influence of sensor configuration and flight altitude on FC estimates as evidenced by differences in FC_LOn_ and FC_LOff_ within conifer plots was 17% (σ = 7%). Largest differences between leaf-on vs. leaf-off lidar FC estimates occurred within deciduous simple plots (D = 32%, σ = 9%).

**Table 6:** Fractional cover results stratified by vegetation type.

Dominate Vegetation Type	Mean Measured FC	Range Measured FC	Leaf-Off vs. Measured		Leaf-On vs. Measured		Leaf-On vs. Leaf-Off	
			*D*	σ (*D*)	*D*	σ (*D*)	*D*	σ (*D*)
Deciduous Compound	0.79	0.69–0.92	−0.19	0.10	0.04^A^	0.04	0.25^A^	0.11
Deciduous Simple	0.81	0.60–0.92	−0.24	0.10	0.07^B^	0.05	0.32^B^	0.09
Mixed	0.84	0.80–0.91	−0.08	0.10	0.10^AB^	0.04	0.18^A^	0.08
Conifer	0.81	0.73–0.89	−0.06	0.08	0.11^B^	0.02	0.17^A^	0.07

Differences between measured fractional cover (FC) and lidar FC estimates (lidar – measured). ANOVA was not run on leaf-off FC estimates as distributions deviated significantly from normal.

Superscripts (A,B,C) identify post-hoc Tukey test results. Means with the same superscripted letter are not statistically different (P = 0.05).

### Laser Pulse Penetration Throughout the Canopy - Influences of Vegetation Condition and Type

Differences in laser pulse penetration depth, demonstrated by both the spread and allocation of lidar point clouds throughout the canopy influenced the accuracy of lidar estimates of canopy height and fractional cover. The overall spread of lidar point clouds, as expressed by the coefficient of variation (*Cv*), varied according to both vegetation condition and type. *Cv* describes the dispersion of returns throughout the canopy with increased dispersion represented by a greater % *Cv* (Table 7). In leafless canopies, the average spread of laser pulse returns (*Cv*_mean_) was 6.9% (σ = 7.3%) greater than the spread of returns in leaf-on canopies. Differences were more pronounced when compared within plots of different leaf structure types. In leafless canopies, laser pulse returns were most spread out within deciduous compound plots (*Cv*_mean_ = 49.4%), compared to a smaller spread in plots dominated by deciduous simple leaved species (*Cv*_mean_ = 39.0%) and conifers (*Cv*_mean_ = 28.4%). A similar pattern (with a lower average *Cv* value) was also observed during leaf-on conditions where increased spread of returns occurs within plots dominated by deciduous compound leaved species (*Cv*_mean_ = 38.0%) compared to plots dominated by deciduous simple leaved species (*Cv*_mean_ = 31.8%) and conifers (*Cv*_mean_ = 27.3%). Observed differences between leaf-on and leaf-off *Cv* values in conifer plots were small (D = 1.1%) whereas largest differences between leaf-off and leaf-on *Cv* values were observed in deciduous compound plots (D = 11.4%).

**Table 7:** Average coefficient of variation (*Cv*) values and associated standard deviation of mean *Cv* for non-ground leaf-off and leaf-on lidar data within all plots and plots stratified by vegetation type.

	Leaf- Off		Leaf-On	
	*D Cv (%)*	σ *Cv (%)*	*D Cv (%)*	σ *Cv (%)*
**All Plots**	37.8 **	10.6	31.0	8.9
**Compound**	49.4^A^ **	9.1	38.0^A^	9.8
**Simple**	39.0^B^ **	9.0	31.8^AB^	7.6
**Mixed**	33.9 **	9.5	27.4^B^	9.0
**Conifer**	28.4^C^	5.6	27.3^B^	7.3

Superscripts (A,B,C) identify post-hoc Tukey test results. Means with the same superscripted letter are not statistically different (P = 0.05). Within each row, leaf-off Cv values that are starred (**) are statistically different when compared to leaf-on cv values for that row (all plots or vegetation type). Statistical differences are not detected between leaf-on and leaf-off Cv values within conifer plots (p<0.05).

**Figure 4:**
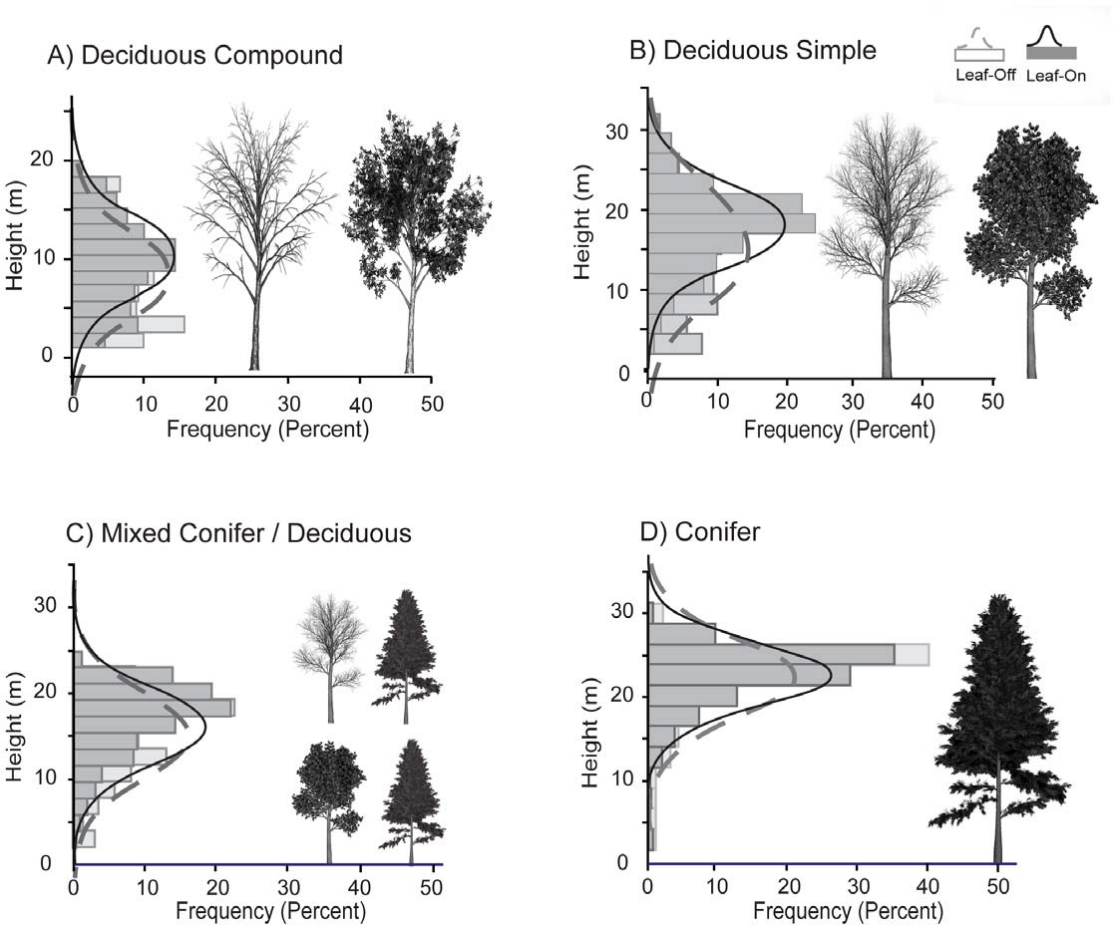
Example distributions of canopy laser pulse returns in leaf-off compared to leaf-on conditions in an A) deciduous compound and B) deciduous simple C) mixed conifer/deciduous and D) conifer plots. Example distributions are representative of conditions observed within all plots for each vegetation type.

While *Cv* values demonstrate differences in overall spread of reflections throughout the canopy, mean differences between leaf-on and leaf-off decile and quartile percentile values and example plot histograms of laser pulse frequency distributions demonstrate differences in reflection allocation at the top, within and at the base of the canopy (Figure 4, Table 8). At the top of the canopy (represented by the top 25% of the lidar distribution), within conifer, simple leaved and mixed plots, pulse returns were triggered close to stem apexes in both leaf-on and leaf-off conditions, yielding small differences in pulse penetration between datasets (−0.2–0.6 m on average, Table 8). However, within deciduous compound plots, laser pulses were triggered 1.0–1.4 m further in leafless compared to leaf-on conditions before eliciting a response. This difference was statistically significant compared to top of the canopy pulse penetration within other vegetation types, including conifer plots, and thus can be attributed to differences in vegetation structure. In all plots, at the bottom of the canopy (represented by the bottom 25% of the distribution), differences in pulse penetration depth within leafless compared to leaf-on canopies were larger compared to the top of the canopy, where in leafless canopies laser pulses were triggered further towards the ground by 1.4–2.6 m on average. Bottom of the canopy penetration depth was also more variable compared to the top, as evidenced by larger standard deviation of mean difference values, which ranged from 3.3–3.5 m on average. Bottom of the canopy differences in pulse penetration were likely caused by a combination of sensor and vegetation effects, as no statistical differences between vegetation types were detected.

**Table 8:** Mean differences (*D*, leaf-on lidar minus leaf-off lidar) between leaf-on and leaf-off lidar percentile height values and associated standard deviation (σ) of mean differences in meters.

	All Plots		Deciduous Simple		Deciduous Compound		Conifer Needle		Mixed	
	*D*	σ (*D*)	*D*	σ (*D*)	*D*	σ (*D*)	*D*	σ (*D*)	*D*	σ (*D*)
*h99*	0.49^A^	0.72	0.59^A^	0.60	**1.07^A^	0.61	0.26	0.89	0.16^A^	0.69
*h95*	0.46^A^	0.74	0.44^A^	0.51	**1.25^A^	1.12	0.15	0.75	0.19^A^	0.51
*h90*	0.38^A^	0.82	0.32^A^	0.63	**1.14^A^	1.20	0.04	0.87	0.21^A^	0.64
*h75*	0.45^A^	0.97	0.31^A^	0.59	**1.37	1.41	−0.17	1.09	0.40^A^	0.85
*h50*	0.59^A^	1.55	0.50	1.28	1.81	1.39	−0.75	1.99	0.64	1.40
*h25*	1.36^B^	3.48	1.40^B^	3.72	3.15^B^	1.82	−1.76	4.93	1.45^B^	2.36
*h10*	2.59	3.34	3.00	3.23	3.16	2.54	1.09	4.91	2.28	3.13

ANOVA was not run on 10^th^ percentile (*h_10_*) values due to nonparametric distribution in leaf-off conditions.

Superscripts (A,B,C) identify post-hoc Tukey test results. Means with the same superscripted letter are not statistically different (P = 0.05). Within each row, values that are starred (**) are statistically different from other values at that percentile within that row.

Histograms of laser pulse return frequency within varying height bins illustrate reflection allocation throughout the canopy (Fig. 4). In conifer and mixed plots, a larger number of returns are reflected within the top of the canopy (>20 m) compared to the bottom of the canopy (<10 m, Fig. 4C, 4D). In these plots, returns towards the bottom of the canopy represented less than 10% of all returns in the distribution in both leaf-on and leaf-off conditions. In contrast, in deciduous simple plots, the number of returns at the top of the canopy was similar to the number of returns towards the bottom of the canopy. In these plots, a larger proportion of returns were located within the upper middle of the canopy in leaf-on conditions. Whereas, in leaf-off conditions more returns were allocated towards the bottom of the canopy yet the top of the canopy was still well-represented by the lidar point cloud distribution (Fig. 4B). Interestingly, in both leaf-on and leaf-off conditions pulse returns were more evenly distributed throughout deciduous compound canopies compared to other plots as represented by a smaller and more uniform frequency of reflections within each height bin within the distribution (Fig. 4A).

## Discussion

Within forested riparian buffers of diverse vegetation structure and composition, lidar flown in leaf-off conditions is sufficient for estimating average canopy height and can be considered for estimating fractional canopy cover within mixed and conifer forests. Furthermore, differences in pulse penetration throughout the canopy as influenced by both vegetation condition (leaf-on vs. leaf-off) and vegetation type (leaf and branch structure) can account for variable relationship strength between lidar estimates and plot measurements.

### Average Canopy Height Estimates

Lidar percentile (*h*_70_) values best approximate measured plot average canopy height (H) during both leaf-on and leaf-off conditions and within plots of varying vegetation types compared to CHM methods. These results are similar to those of Hawbaker et al. [39] who found that upper height percentile lidar estimates most closely approximated *in situ* H measurementss. Interpolated CHM values underestimate top of the canopy heights by a comparatively larger margin (Fig. 2). CHM underestimation of canopy height has been observed in other studies [e.g. 56]. For example, Hopkinson et al. [56] found that interpolated lidar CHM's underestimated tree and shrub height by 0.3 m–1.1 m on average representing 3 to 30% of average vegetation heights in the areas studies. Similarly, Gaveau and Hill [57] found lidar CHMs to underestimate shrub and tree height by 1–2 m respectively. This underestimation of H by lidar CHMs can be attributed to both raster creation methods and resolution. For example, IDW interpolation methods that maintain near-point integrity can be more sensitive to canopy gaps, which contain tall saplings, and suppressed canopy vegetation which can in turn negatively bias height values. Biased CHM estimates of canopy height has also been attributed to laser pulse returns missing tree crown apexes in conjunction with interpolation method smoothing effects which propagate bias throughout the CHM [57]. In this study, a 1.8 m resolution grid was produced to maintain lidar data (1.4 m point spacing) near-point integrity. However, a higher resolution grid generated from higher resolution lidar data will likely improve CHM estimates. Non-interpolated CHM methods (using the max height value) produced *H* values that were closer to *h*_70_ yet still under and overestimated H by a larger margin compared to *h*_70_ values. However, if raster methods are required, non-interpolated CHM max height values provide a better representation of top of the canopy conditions compared to interpolated raster methods.

Lidar percentile values estimate plot measurements well in both leaf-off and leaf-on conditions however are influenced by vegetation type. Differences are largest in deciduous compound plots (compared to plots dominated by other vegetation types) where laser pulse returns sink further in leafless compared to leaf-on canopies before triggering a response (Fig. 4, Table 8). Thus, in deciduous compound plots, the overall distribution of returns is lower relative to the actual top of the canopy, and a value higher within the lidar point cloud distribution more closely represents measured values. For example, the 70^th^ percentile underestimates measured plot height in deciduous compound plots, whereas it overestimates average height in other plots (Table 4).

The similarity of lidar top of the canopy percentile estimates in leaf-off compared to leaf-on conditions within deciduous plots contradicts the expectation that laser pulses will reflect lower down within leafless canopies due to decreased leaf biomass. It is possible that in some plots top of the canopy responses may have reflected from a small proportion of conifer stems found in some deciduous plots (composing 10% or less of all stems on average). However, other studies demonstrate that for some deciduous species, laser pulse returns reflect from the top of the canopy, even in leaf-off conditions [40]. For example, Brandtberg et al. [42] found no significant underestimation of canopy heights during leaf-off conditions within Eastern North American deciduous forests. Orka et al. [41] found birch species in Scandinavian forests elicited returns that are, on average, higher in leafless compared to leaf-on conditions. Thus, it is reasonable to conclude that in leaf-off conditions, dense riparian branch structure yields top of the canopy returns that are sufficient to estimate measured average canopy height.

### Fractional Canopy Cover

Differences were observed between leaf-off and leaf-on lidar FC estimates and measured FC estimates. Leaf-off lidar FC (FC_Loff_) underestimates FC by 19–24% in deciduous canopies, however, represents measured FC (FC_DHP_) well within conifer and mixed canopies. Underestimation of FC in deciduous canopies is likely due to decreased leafy biomass yielding increased ground penetration. Interestingly, while a large underestimation of FC_DHP_ might be expected within mixed plots (due to deciduous vegetation), observed differences are small and similar to differences detected when using leaf-on lidar data. It is likely that a balance between decreased leafy biomass that facilitates ground penetration and dense needle cover that ensures within canopy responses (as observed in plot histograms, Fig. 4C) yields close estimates of FC_DHP_. When compared to leaf-off lidar FC estimates, leaf-on lidar estimates relate well to but also overestimate FC_DHP_ within all vegetation types by 4–11%. Overestimation of FC is likely due to limited ground penetration, which is particularly evident in lidar point cloud histograms within conifer plots where few returns are elicited at the bottom of the point cloud distribution (Fig. 4D); subsequently FC is overestimated by the largest margin in conifer plots. Within deciduous simple leaved plots, a similar pattern of FC_Lon_ overestimation occurs. In contrast, FC_DHP_ is most closely estimated in deciduous compound leaved plots where increased pulse penetration is observed throughout the canopy (Fig. 4A).

Overestimation of fractional cover by lidar can be attributed in part to canopy density, composition and structure, which can limit pulse ground penetration [31], [56]. However, over and underestimation of FC by lidar can also be attributed to flight configuration. Higher flight altitudes, and associated changes in pulse footprint and peak pulse power have been shown to yield increased single returns and associated decreased second/multiple returns per pulse [58]–[60]. In some forest types, higher altitude collections may yield lidar overestimation of FC [61] due to a wider footprint which yields reduced ground response [60]. However, flight configuration has also been associated with underestimated FC due to reduced vegetation response and increased ground penetration [62]. In this study, within conifer plots, FC_Loff_ estimates are 20% lower than FC_Lon_ on average. However, it is important to note that in these same plots, FC_Loff_ and FC_Lon_ estimates are still within +/−10% of measured FC. Similarly, while vegetation type can influence lidar FC estimate accuracy [38], in this study, differences are less than the errors associated with digital hemispherical photography methods [55]. Therefore, as observed within the Spring Creek watershed, the degree of deviation of leaf-on (all plots) and leaf-off (mixed and conifer plots) lidar FC estimates from measured FC values is within the estimated error of DHP methods and thus likely acceptable for broadscale mapping purposes [27]. For example leaf-off lidar may be sufficient for detecting the 30% tree cover threshold often used to classify forest. However, it is also important to note that the range of variability of canopy cover sampled is narrow (60–90% cover) given dense riparian vegetation throughout the study watershed. Thus lidar estimates might be more variable within different vegetation types given lower density canopies.

### Vegetation Type & Lidar Flight/Configuration Influences on Lidar Estimates

Throughout the canopy, pulse penetration depth varies with both vegetation condition and type influencing lidar estimates of plot measurements. Variable penetration depth may be attributed to altitude differences between the two lidar surveys given that emitted light energy will attenuate and spread as it travels towards the earth's surface and through the canopy [58], [62]. However, these differences, particularly at the top of the canopy, are likely small [58], [60], [61]. Leaf-on and leaf-off top of the canopy height estimates (e.g. *h*_70_) and the overall spread of laser pulse returns (*Cv*) are similar within conifer plots. For example, throughout the top 25% of the lidar point cloud distribution, differences between leaf-on and leaf-off laser pulse penetration (represented by mean differences of percentile height values) range from −0.2 m to 0.3 m (Table 8). Whereas differences observed within the top 25% of deciduous compound plots are larger and statistically significant when compared to conifer plots. The overall spread of laser pulse returns (represented by coefficient of variation) in leaf-on compared to leaf-off conditions is also within 1% in conifer plots (Table 7). In comparison, larger and statistically significant differences are observed within deciduous simple and deciduous compound plots. These results support findings in other studies. For example, Goodwin [58] found no significant difference between canopy height profiles derived from lidar flown at altitudes ranging from 1,000–3,000 m. Hopkinson [60] and Naesset [59] demonstrated that differences in altitude yield a small underestimation of maximum canopy height (0.2 m to 0.6 m on average), whereas Naesset [61] determined lidar estimates of forest biophysical conditions were robust regardless of flight altitude.

Therefore, differences in top of the canopy penetration depth are likely due to a combination of species-specific differences in canopy architecture (patterns of lateral branching and associated leaf angle and geometry) and leaf condition (leaf-on vs. leaf off). Specifically, in the Spring Creek watershed, largest differences are observed within deciduous compound plots. The spreading and open branch structure of vegetation within these plots (e.g. Black Walnut, *J. nigra*) most likely facilitates increased pulse penetration in both leaf-off and leaf-on conditions. Variation in leaf and branch structure influences incident light energy reflection, transmission and absorption within canopies [63], maximizing light intake and ecosystem productivity [64]. Thus, it is not surprising that this variability can influence both the spread and allocation of laser pulses in leaf-on and leaf-off canopies [40]–[44], [53], [65], [66]. Orka (2010) found top of the canopy lidar height estimates to be lower in leaf-off compared to leaf-on conditions for Aspen species whereas estimates are higher in leaf-off compared to leaf-on conditions for Birch species.

Interestingly, laser pulse penetration at the top of the canopy (used to estimate canopy height) is less variable (represented by a smaller standard deviation of mean differences between leaf-on and leaf-off lidar percentile values) compared to penetration values observed towards the bottom of the canopy (Table 8). Given this bottom of the canopy variability in penetration depth, it is expected that within canopy estimates of vegetation structure (e.g. canopy base height, sapling and understory cover) might yield a weaker correlation with measured values compared to top of the canopy height estimates. This decreased strength in within canopy lidar estimates has been observed when using lidar to estimate canopy base height (unpublished data) and understory canopy structure [67]. Bottom of the canopy differences in pulse penetration are likely a combined effect of system configuration and vegetation structure. Increased flight altitude, not only yields fewer multiple returns [58], but also can yield increased variability of within canopy pulse penetration depth [40], [62]. However, this variability could also be attributed to branch volume and associated leaf area per branch which often increases lower in the canopy to maximize light absorption [68], [69]. Thus, differences observed within canopy pulse penetration depth may also represent naturally occurring variability in leaf and branch structure.

## Conclusions

The implications of this work for the study of riparian forests are as follows:

1. Given diverse top of the canopy vertical structure and 1.4 m lidar point spacing, lidar CHM methods underestimate and deviate further from measured average canopy heights compared to lidar percentile methods. Lidar percentile methods to predict canopy height may be preferred for broadscale mapping purposes.
2. Lidar percentile estimates are similar to measured H within deciduous simple, conifer and mixed plots during leaf-on and leaf-off conditions, however, lidar percentile methods underestimate H in deciduous compound canopies. Further, field validation may be needed to assess potential error when using lidar to sample vegetation over broad geographic extents.
3. Lidar estimates of fractional cover are within 10% of measured in all plots during leaf-on periods, and in mixed deciduous/conifer canopies during leaf-off periods, but are greatly underestimated during leaf-off periods in deciduous canopies; leaf-on lidar therefore may be preferred for fractional cover estimates.
4. Depth of penetration lower in the canopy is more variable compared to top of the canopy penetration. Differences in penetration depth will likely influence accuracy of within canopy vegetation structure estimates such as canopy base height and understory cover.
5. Sensor and flight altitude effects observed within conifer plots account for small differences (less than 0.5 m) in top of the canopy penetration. These effects may be more pronounced within and at the bottom of the canopy.

This study provides a viable rationale for further examination of lidar datasets that are not optimized for vegetation detection. It furthermore demonstrates the use of lidar to reliably estimate forested riparian buffer structure across a watershed given varying vegetation conditions. Lidar data surveyed during leaf-off conditions are sufficient to estimate forested riparian buffer canopy height and may be sufficient to estimate fractional cover in mixed forests when leaf-on datasets are not available. However, differences in laser pulse penetration in leaf-off compared to leaf-on conditions and within varying vegetation types will influence lidar estimate accuracy. Therefore, field calibration that considers vegetation type and structure may be required to both select best lidar methods and calibrate lidar models when measuring height for some purposes such as growth detection. However, there is great potential for existing leaf-off lidar data to support a host of broadscale inventory, analysis and change detection mapping purposes including detection of riparian vegetation height classes, assessment of forested riparian buffer vegetation density and biomass and determination of forested buffer width. Broadscale mapping of riparian buffer structure efforts is essential to assess buffer and associated stream and wildlife population integrity in support of ecological monitoring that assesses disturbance impacts and associated restoration efforts.

## References

[1] Naiman RJ, Decamps H, Pollock M (1993) The Role of Riparian Corridors in Maintaining Regional Biodiversity. Ecological Applications 3: 209–212. 27759328 10.2307/1941822

[2] Naiman RJ, Decamps H (1997) The ecology of interfaces: Riparian zones. Annual Review of Ecology and Systematics 28: 621–658.

[3] Perry CD, Vellidis G, Lowrance R, Thomas DL (1999) Watershed-scale water quality impacts of riparian forest management. Journal of Water Resources Planning and Management-Asce 125: 117–125.

[4] Snyder MN, Goetz SJ, Wright RK (2005) Stream health rankings predicted by satellite derived land cover metrics. Journal of the American Water Resources Association 41: 659–677.

[5] Mayer PM, Steven K, Reynolds J, Canfield TJ (2005) Riparian Buffer Width, Vegetative Cover, and Nitrogen Removal Effectiveness: A Review of Current Science and Regulations. US EPA, Office of Research and Development. National Risk Management Research Laboratory. 40 p.

[6] Lowrance R, Altier LS, Newbold JD, Schnabel RR, Groffman PM, et al (1997) Water quality functions of Riparian forest buffers in Chesapeake Bay watersheds. Environmental Management 21: 687–712. 9236284 10.1007/s002679900060

[7] Sutton AJ, Fisher TR, Gustafson AB (2010) Effects of Restored Stream Buffers on Water Quality in Non-tidal Streams in the Choptank River Basin. Water Air and Soil Pollution 208: 101–118.

[8] Speiran GK (2010) Effects of Groundwater-Flow Paths On Nitrate Concentrations Across Two Riparian Forest Corridors1. Journal of the American Water Resources Association 46: 246–260.

[9] Hall R, Watkins R, Heggem D, Jones K, Kaufmann P, et al (2009) Quantifying structural physical habitat attributes using LIDAR and hyperspectral imagery. Environmental Monitoring and Assessment 159: 63–83. 19165614 10.1007/s10661-008-0613-y

[10] Arroyo LA, Johansen K, Armston J, Phinn S (2010) Integration of LiDAR and QuickBird imagery for mapping riparian biophysical parameters and land cover types in Australian tropical savannas. Forest Ecology and Management 259: 598–606.

[11] Bain MB, Harig AL, Loucks DP, Goforth RR, Mills KE (2000) Aquatic ecosystem protection and restoration: advances in methods for assessment and evaluation. Environmental Science and Policy Environmental Science and Policy 3: 89–98.

[12] Seavy NE, Viers JH, Wood JK (2009) Riparian bird response to vegetation structure: a multiscale analysis using LiDAR measurements of canopy height. Ecological Applications 19: 1848–1857. 19831074 10.1890/08-1124.1

[13] Smith TA, Osmond DL, Moorman CE, Stucky JM, Gilliam JW (2008) Effect of vegetation management on bird habitat in Riparian buffer zones. Southeastern Naturalist 7: 277–288.

[14] Bradbury RB, Hill RA, Mason DC, Hinsley SA, Wilson JD, et al (2005) Modelling relationships between birds and vegetation structure using airborne LiDAR data: a review with case studies from agricultural and woodland environments. Ibis 147: 443–452.

[15] DeWalle DR (2010) Modeling Stream Shade: Riparian Buffer Height and Density as Important as Buffer Width1. Journal of the American Water Resources Association 46: 323–333.

[16] Broadmeadow S, Nisbet TR (2004) The effects of riparian forest management on the freshwater environment: a literature review of best management practice. Hydrology and Earth System Sciences 8: 286–305.

[17] Brooks R, McKenney-Easterling M, Brinson M, Rheinhardt R, Havens K, et al (2009) A Stream-Wetland-Riparian (SWR) index for assessing condition of aquatic ecosystems in small watersheds along the Atlantic slope of the eastern US. Environmental Monitoring and Assessment 150: 101–117. 19082749 10.1007/s10661-008-0673-z

[18] Vidon P (2010) Riparian zone management and environmental quality: a multi-contaminant challenge. Hydrological Processes 24: 1532–1535.

[19] Goetz SJ (2006) Remote sensing of riparian buffers: Past progress and future prospects (vol 1, pg 133, 2006). Journal of the American Water Resources Association 42: 806–806.

[20] Petersen RC (1992) The RCE - A Riparian, Channel, and Environmental Inventory for Small Streams in the Agricultural Landscape. Freshwater Biology 27: 295–306.

[21] Schuft MJ, Moser TJ, Wigington PJ, Stevens DL, McAllister LS, et al (1999) Development of landscape metrics for characterizing riparian-stream networks. Photogrammetric Engineering and Remote Sensing 65: 1157–1167.

[22] Snyder CD, Young JA, Villella R, Lemarie DP (2003) Influences of upland and riparian land use patterns on stream biotic integrity. Landscape Ecology 18: 647–664.

[23] Claggett PR, Okay JA, Stehman SV (2010) Monitoring Regional Riparian Forest Cover Change Using Stratified Sampling and Multiresolution Imagery. Journal of the American Water Resources Association 46: 334–343.

[24] Lattin PD, Wigington PJ, Moser TJ, Peniston BE, Lindeman DR, et al (2004) Influence of remote sensing imagery source on quantification of Riparian land cover and use. Journal of the American Water Resources Association 40: 215–227.

[25] Vierling KT, Vierling LA, Gould WA, Martinuzzi S, Clawges RM (2008) Lidar: shedding new light on habitat characterization and modeling. Frontiers in Ecology and the Environment 6: 90–98.

[26] Crew R (2007) Riparian Forest and Wetland Inventory and Analysis for the Chesapeake Bay Watershed. University Park: The Pennsylvania State University. 167 p.

[27] Smith AMS, Falkowski MJ, Hudak AT, Evans JS, Robinson AP, et al (2009) A cross-comparison of field, spectral, and lidar estimates of forest canopy cover. Canadian Journal of Remote Sensing 35: 447–459.

[28] Hopkinson C, Chasmer L (2009) Testing LiDAR models of fractional cover across multiple forest ecozones. Remote Sensing of Environment 113: 275–288.

[29] Hopkinson C, Chasmer L, Lim K, Treitz P, Creed I (2006) Towards a universal lidar canopy height indicator. Canadian Journal of Remote Sensing 32: 139–152.

[30] Morsdorf F, Koetz B, Meier E, Itten KI, Allgower B (2005) The Potential of Discrete Return, Small Footprint Airborne Laser Scanning Data for Vegetation Density Estimation. Proceedings of ISPRS WG III/3, III/4 3, 12–14. Netherlands.

[31] Morsdorf F, Kötz B, Meier E, Itten KI, Allgöwer B (2006) Estimation of LAI and fractional cover from small footprint airborne laser scanning data based on gap fraction. Remote Sensing of Environment 104: 50–61.

[32] Hill RA, Broughton RK (2009) Mapping the understorey of deciduous woodland from leaf-on and leaf-off airborne LiDAR data: A case study in lowland Britain. Isprs Journal of Photogrammetry and Remote Sensing 64: 223–233.

[33] Naesset E (2002) Predicting forest stand characteristics with airborne scanning laser using a practical two-stage procedure and field data. Remote Sensing of Environment 80: 88–99.

[34] Naesset E (1997) Estimating timber volume of forest stands using airborne laser scanner data. Remote Sensing of Environment 61: 246–253.

[35] Naesset E, Gobakken T, Nelson R (2011). Sampling and mapping forest volume and biomass using airborne lidars; 2006 2007. Monterey, CA. pp. 297–301.

[36] Naesset E, Okland T (2002) Estimating tree height and tree crown properties using airborne scanning laser in a boreal nature reserve. Remote Sensing of Environment 79: 105–115.

[37] Johansen K, Phinn S, Witte C (2010) Mapping of riparian zone attributes using discrete return LiDAR, QuickBird and SPOT-5 imagery: Assessing accuracy and costs. Remote Sensing of Environment 114: 2679–2691.

[38] Chasmer L, Hopkinson C, Treitz P, McCaughey H, Barr A, et al (2008) A lidar-based hierarchical approach for assessing MODIS fPAR. Remote Sensing of Environment 112: 4344–4357.

[39] Hawbaker TJ, Gobakken T, Lesak A, Tromborg E, Contrucci K, et al (2010) Light Detection and Ranging-Based Measures of Mixed Hardwood Forest Structure. Forest Science 56: 313–326.

[40] Naesset E (2005) Assessing sensor effects and effects of leaf-off and leaf-on canopy conditions on biophysical stand properties derived from small-footprint airborne laser data. Remote Sensing of Environment 98: 356–370.

[41] Orka HO, Naesset E, Bollandsas OM (2010) Effects of different sensors and leaf-on and leaf-off canopy conditions on echo distributions and individual tree properties derived from airborne laser scanning. Remote Sensing of Environment 114: 1445–1461.

[42] Brandtberg T, Warner TA, Landenberger RE, McGraw JB (2003) Detection and analysis of individual leaf-off tree crowns in small footprint, high sampling density lidar data from the eastern deciduous forest in North America. Remote Sensing of Environment 85: 290–303.

[43] Brandtberg T (2007) Classifying individual tree species under leaf-off and leaf-on conditions using airborne lidar. Isprs Journal of Photogrammetry and Remote Sensing 61: 325–340.

[44] Kim S, McGaughey RJ, Andersen HE, Schreuder G (2009) Tree species differentiation using intensity data derived from leaf-on and leaf-off airborne laser scanner data. Remote Sensing of Environment 113: 1575–1586.

[45] Song J-H, Soo-Hee-Han, Yu K, Kim Y-I (2002) Assessing the Possibility of Land-Cover Classification Using LiDAR Intensity Data. In: III IC, editor. Photogrammetric Computer Vision Symposium 2002: ISPRS Commission III. pp. 259–263.

[46] Anderson ES, Thompson JA, Austin RE (2005) LIDAR density and linear interpolator effects on elevation estimates. International Journal of Remote Sensing 26: 3889–3900.

[47] Nilsson M (1996) Estimation of tree heights and stand volume using an airborne lidar system. Remote Sensing of Environment 56: 1–7.

[48] Farve R (2010) Evaluation of Laser Rangefinders. US Forest Service. 60 p.

[49] Warman L, Moles AT, Edwards W (2011) Not so simple after all: searching for ecological advantages of compound leaves. Oikos 120: 813–821.

[50] Niinemets U (1998) Are compound-leaved woody species inherently shade-intolerant? An analysis of species ecological requirements and foliar support costs. Plant Ecology 134: 1–11.

[51] Malhado ACM, Whittaker RJ, Malhi Y, Ladle RJ, ter Steege H, et al (2010) Are compound leaves an adaptation to seasonal drought or to rapid growth? Evidence from the Amazon rain forest. Global Ecology and Biogeography 19: 852–862.

[52] Ustin SL, Martens SN, Vanderbilt VC (1991) Canopy architecture of a walnut orchard. Geoscience and Remote Sensing, IEEE Transactions on 29: 843–851.

[53] Korpela I, Orka HO, Maltamo M, Tokola T, Hyyppa J (2010) Tree Species Classification Using Airborne LiDAR - Effects of Stand and Tree Parameters, Downsizing of Training Set, Intensity Normalization, and Sensor Type. Silva Fennica 44: 319–339.

[54] Leblanc SG, Chen JM, Fernandes R, Deering DW, Conley A (2005) Methodology comparison for canopy structure parameters extraction from digital hemispherical photography in boreal forests. Agricultural and Forest Meteorology 129: 187–207.

[55] Chen JM, Govind A, Sonnentag O, Zhang YQ, Barr A, et al (2006) Leaf area index measurements at Fluxnet-Canada forest sites. Agricultural and Forest Meteorology 140: 257–268.

[56] Hopkinson C, Chasmer LE, Sass G, Creed IF, Sitar M, et al (2005) Vegetation class dependent errors in lidar ground elevation and canopy height estimates in a boreal wetland environment. Canadian Journal of Remote Sensing 31: 191–206.

[57] Gaveau DLA, Hill RA (2003) Quantifying canopy height underestimation by laser pulse penetration in small-footprint airborne laser scanning data. Canadian Journal of Remote Sensing 650–657.

[58] Goodwin NR, Coops NC, Culvenor DS (2006) Assessment of forest structure with airborne LiDAR and the effects of platform altitude. Remote Sensing of Environment 103: 140–152.

[59] Naesset E (2009) Effects of different sensors, flying altitudes, and pulse repetition frequencies on forest canopy metrics and biophysical stand properties derived from small-footprint airborne laser data. Remote Sensing of Environment 113: 148–159.

[60] Hopkinson C (2007) The influence of flying altitude, beam divergence, and pulse repetition frequency on laser pulse return intensity and canopy frequency distribution. Canadian Journal of Remote Sensing 33: 312–324.

[61] Naesset E (2004) Effects of different flying altitudes on biophysical stand properties estimated from canopy height and density measured with a small-footprint airborne scanning laser. Remote Sensing of Environment 91: 243–255.

[62] Morsdorf F, Frey O, Meier E, Itten KI, Allgöwer B (2008) Assessment of the influence of flying altitude and scan angle on biophysical vegetation products derived from airborne laser scanning. International Journal of Remote Sensing 29: 1387–1406.

[63] Hyer EJ, Goetz SJ (2004) Comparison and sensitivity analysis of instruments and radiometric methods for LAI estimation: assessments from a boreal forest site. Agricultural and Forest Meteorology 122: 157–174.

[64] Ishii HT, Tanabe S, Hiura T (2004) Exploring the relationships among canopy structure, stand productivity, and biodiversity of temperature forest ecosystems. Forest Science 50: 342–355.

[65] Magnussen S, Naesset E, Gobakken T (2010) Reliability of LiDAR derived predictors of forest inventory attributes: A case study with Norway spruce. Remote Sensing of Environment 114: 700–712.

[66] Orka HO, Naesset E, Bollandsas OM (2009) Classifying species of individual trees by intensity and structure features derived from airborne laser scanner data. Remote Sensing of Environment 113: 1163–1174.

[67] Korpela I, Hovi A, Morsdorf F (2011) Mapping Understory Trees Using Airborne Discrete-Return Lidar Data. International Society for Photogrammetry and Remote Sensing. Hanover, Germany: ISPRS. pp. 6.

[68] Valladares F, Niinemets U (2007) The Architecture of plant crowns: form design rules to light capture and performance. In: Pugnaire F, Valladares F, editors. Functional Plant Ecology. New York: Yaylor & Francis. pp. 101–149.

[69] Sumida A, Komiyama A (1997) Crown spread patterns for five deciduous broad-leaved woody species: Ecological significance of the retention patterns of larger branches. Annals of Botany 80: 759–766.

